# Dr. J. Austin Dunn

**Published:** 1894-11

**Authors:** 


Dr. J. Austin Dunn of Chicago has reduced the price of his Syringe with one Iridio-Platinum needle to $1.50. The Iridio-Platinum needle is something the dentists have always wanted but could not procure, and is a step in advance. The Iridium makes the needle stiff, so that it will wear smooth and is more lasting and durable. Another advantage is, that it can be held in the flame of the spirit lamp and cleaned by burning out the debris without danger of softening it. The price for extra needles has been fixed at fifty cents, so that they are within the reach of all.

